# Clinical manifestations and severity of cow’s milk anaphylaxis in children

**DOI:** 10.1186/2045-7022-1-S1-P47

**Published:** 2011-08-12

**Authors:** Zahra Pourpak, Pegah Teymourpour, Saeideh Barzegar, Raheleh Shokouhi, Mohammadreza Fazlollahi, Mostafa Moin

**Affiliations:** 1 Immunology,Asthma and Allergy Research Institute,Tehran University of Medical Sciences, Tehran, Islamic Republic of Iran

## Background

Anaphylaxis is a potentially fatal allergic reaction which is rapid in onset. Cow's milk is the most common allergen which can trigger anaphylaxis in Iranian children.The aim of this study is to describe the clinical features and severity of cow's milk anaphylaxis in children.

## Methods

In this study all children(<18 years old) with suspicious history of cow's milk anaphylaxis who had been referred to IAARI(Immunology,Asthma and Allergy Research Institute)during 2005(JAN)—2010(SEP) were considered.A detailed questionnaire was fulfilled for each patient. A specific severity grading for anaphylaxis with five levels was also used. Skin Prick Test(SPT) with allergen extract(cow's milk) was performed at least 4—6 weeks after patient's last anaphylaxis attack. Cow's milk specific IgE and total IgE level were measured by Immunocap system.Patients with clear history and one positive laboratory test and patients with both positive laboratory tests(SPT and cow's milk specific IgE)were included.

## Results

Among 49 patients ,29(59.2‰) were male and the rest were female.Patients' mean age at the time of first anaphylactic attack was 5.7±4.3 months.The percentages of severity grades 1- 5 were 2‰,6.1‰,18.4‰,69.4‰,4.1‰ respectively. Most common clinical manifestations were in descending order as: Cutaneous98‰(urticaria,periorbital oedema,flushing),Respiratory91.8‰(dyspnea,coughing,wheezing), Gastrointestinal 55.1‰(nausea,vomiting,abdominal pain),Cardiovascular 46.9‰ and Neurologic 46.9‰.Twenty- four patients had positive SPT. Mean total IgE level was 239.6±3.3 (KU/L) and mean cow's milk specific IgE was 19.28±27.2(KU/L).

## Conclusions

Since the patients’mean age at the time of first anaphylactic shock was 5.7±4.3 months, we conclude that cow's milk anaphylaxis may happen very early in life. Most common manifestations are cutaneous and respiratory symptoms. Majority of attacks have been of moderate to relatively severe degree(grades 3 and 4) so equipping the general practitioners and pediatricians with enough knowledge about cow's milk anaphylaxis is needed.

